# Association of vision and hearing impairment and dietary diversity among the oldest old in China: findings from the Chinese longitudinal healthy longevity survey

**DOI:** 10.1186/s12889-024-19482-x

**Published:** 2024-07-25

**Authors:** Xinyue Shen, Xuhao Chen, Xiaohong Chen, Zhidong Li, Junxiong Lin, Haishun Huang, Rui Xie, Yiqing Li, Yingting Zhu, Yehong Zhuo

**Affiliations:** State Key Laboratory of Ophthalmology, Zhongshan Ophthalmic Center, Sun Yat-sen University, Guangdong Provincial Key Laboratory of Ophthalmology Visual Science, Guangdong Provincial Clinical Research Center for Ocular Diseases, Guangzhou, 510060 China

**Keywords:** Visual impairment, Hearing impairment, Dietary diversity, Oldest old, Healthy aging

## Abstract

**Background:**

The presence of sensory impairment among older age cohorts exerts a significant impact on both individuals and society generally. Although the impact of dietary patterns on health is vital across all stages of life, there still a paucity of comprehensive research on the association between dietary variety and sensory impairments.

**Objective:**

To investigate the potential relationship between dietary diversity and the prevalence of visual and hearing impairment or dual sensory impairments (visual and hearing impairment) among the oldest old population.

**Methods:**

This is a cross-sectional study relied on data obtained from the 2018 survey conducted by the Chinese Longitudinal Healthy Longevity Survey (CLHLS). Subjects aged 80 and older with complete vision and hearing data were included in the study. Multivariate logistic regression models were developed to examine the association between dietary components and visual and hearing impairment while controlling for age, gender, socioeconomic demographic factors, living habits, other food habits, and general health status.

**Results:**

The study included 10,093 participants, with an average age of 92.29 ± 7.75 years. Vision and hearing function were assessed based on the ability to distinguish the direction of the break in the circle and the requirement for hearing aids, respectively. Upon controlling for confounding variables, individuals with a greater Dietary Diversity Score (DDS, the number of food groups, range: 1–11) had a reduced likelihood of experiencing visual impairment (odds ratio [OR] = 0.944, 95% confidence interval [CI], 0.915—0.974) and dual sensory impairment (OR = 0.930, 95% CI, 0.905—0.955). In comparison to the low dietary variety group (insufficient dietary diversity, DDS < 4), the high dietary diversity group (sufficient dietary diversity, DDS ≥ 4) exhibited a decreased risk of visual impairment (OR = 0.820, 95% CI, 0.713—0.944) and dual sensory impairment (OR = 0.751, 95% CI, 0.667—0.846). However, no statistically significant correlation was observed between dietary diversity and the presence of only hearing impairment (OR = 0.924, 95% CI, 0.815—1.047) (*P* < 0.05).

**Conclusions and implications:**

The synthesis of research findings suggests that following diverse dietary patterns and healthy nutritional practices may be an effective and affordable way to prevent age-related decline in visual impairment and dual sensory impairment.

## Introduction

According to the World Health Organization (WHO), aging is attributed to the cumulative effects of diverse molecular and cellular damage over time, resulting in a steady decline in physical and cognitive abilities [1]. The increasing global population aging has emerged as a complex public health concern confronting numerous nations, including China. The WHO report indicates a worldwide surge in both the quantity and percentage of older adults globally, with projections suggesting that individuals aged 60 years and above will rise from representing 12% to reaching as high as 22% by the year 2050. Moreover, there is an expected tripling in the number of octogenarians during this timeframe [2]. Based on the standard demographic definition, individuals classified as the "oldest old" are typically aged 80 and above [3]. This particular age group is prone to necessitate increased primary care and medical interventions. Therefore, functional impairments associated with advanced age are expected to have a significant impact on individuals, families and social healthcare systems [4].

Sensory impairments (SI), including vision impairment (VI), hearing impairment (HI), and dual sensory impairment (DSI), are prevalent among the older population globally and have a direct impact on their daily functioning [5, 6]. Specifically, it is projected that a minimum of 6.1 billion individuals will encounter vision impairment [7, 8], while an estimated 2.5 billion people worldwide are expected to experience hearing impairment by the year 2050 [9]. Studies have indicated that sensory impairment negatively affects older individuals' communication and social engagement, which in turn, can further contribute to an elevated risk of cognitive decline, dementia, depression, anxiety, perceived discrimination, and loneliness [10–19]. Most previous studies have predominantly focused on the adverse consequences of visual and hearing impairments, whereas limited studies have investigated the possible risk or protective factors that contribute to the development of sensory impairments.

Several studies have indicated a correlation between physical activity and the severity of vision and hearing impairments. Engaging in physical activity can enhance ocular health, exhibit a negative correlation with auditory impairment, and exert a positive impact on the process of healthy aging [20–22]. Promoting sports among the elderly may be challenging due to their decline in physical function. Compared to other lifestyles, diet holds paramount importance in daily life, particularly for the elderly, as it offers a more convenient means of adjustment and optimization. A healthy and scientific diet pattern, as a modifiable lifestyle factor, has been found beneficial to preventing cardiovascular diseases, promoting excellent physical function, and reducing overall death rates, particularly among the oldest old [23–25]. Consuming a well-balanced and varied diet has been shown to contribute to the prevention of cardiovascular disease, the preservation of optimal physical function, and a notable reduction of all-cause mortality [26]. Therefore, comprehending the interplay between diet-related factors and the health status of older individuals is imperative for preempting the onset and decelerating the progression of age-associated ailments.

In general, the mitigation of sensory impairments is of significant importance in facilitating the older population's ability to maintain a high standard of living and promote healthy aging. The Dietary Diversity score (DDS) serves as a promising indicator of dietary quality and nutritional sufficiency by quantifying the variety of food groups consumed within a specific timeframe. As a proficient and user-friendly tool for assessing dietary quality and nutritional status, DDS has gained widespread usage in numerous studies to evaluate the diet quality and health status of diverse age groups and national populations [27]. Several studies have demonstrated that certain diverse, well-balanced, and nutritious dietary patterns can potentially modulate nutrient sensing pathways, gut microbiota composition, metabolism, and immunity to delay the aging process [28]. Given that sensory dysfunction is a prominent manifestation of aging, it is plausible to hypothesize that dietary diversity will be negatively correlated to vision and hearing impairment. To our knowledge, currently available evidence does not provide substantial support for such a correlation in the oldest old population. Hence, the objective of this study is to investigate the potential correlation between increased dietary diversity and a reduced likelihood of sensory impairments among the oldest old, using a nationally representative sample of the Chinese elderly population.

## Methods

### Data and study participants

The data of this study was based on the Chinese Longitudinal Healthy Longevity Survey (CLHLS), a survey program administered by the Center for Healthy Aging and Development at Peking University. The CLHLS performed eight waves of surveys between 1998 and 2018 (source: https://opendata.pku.edu.cn/). The researchers employed a multistage cluster random sampling technique to select individuals aged 60 years and above from 23 provinces in China, representing approximately 85% of the country's total population. Approximately 67.4% of the overall sample consists of individuals aged 80 years or older [29].

**Fig. 1:**
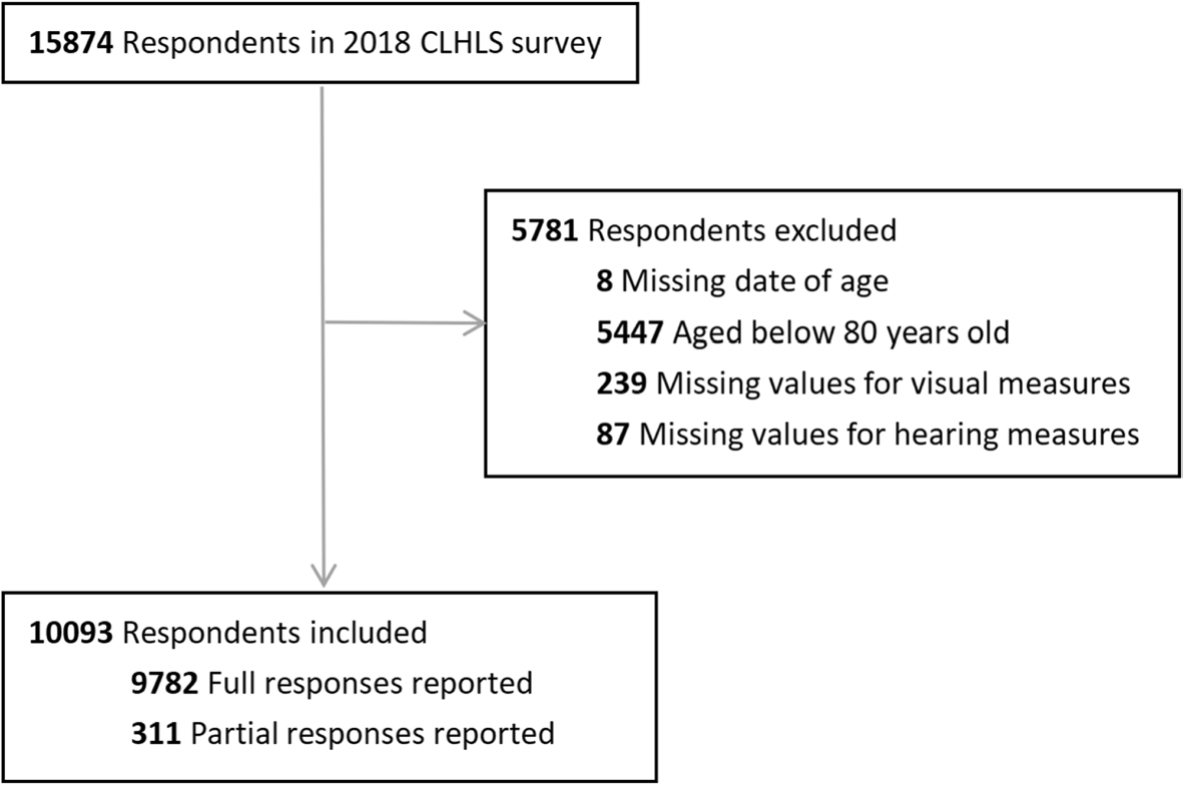
Flow chart of the study sample from the 2018 China health and longevity longitudinal survey (CLHLS)

To examine the relationship between sensory impairments and daily dietary diversity among the oldest old in China, we utilized data from the most recent wave (2018) of the CLHLS, which included a sample size of 15,874 individuals. For analysis, the current cross-sectional study comprised participants aged 80 years and above, referred to as the oldest old. The flow of the inclusion and exclusion criteria is depicted in Fig. 1. 10,093 individuals were eventually enrolled in the cohort.

The project was approved by the Biomedical Ethics Committee of Peking University, China (IRB00001052-13074), and all subjects or their respondents signed written informed consent for baseline and follow-up surveys. The present study entailed a secondary analysis of established data sets and was not subject to additional ethical approval according to the London School of Economics and Political Science research ethics policy and procedures.

## Assessment of sensory impairment

The presence of sensory impairment was determined by assessing the functionality of vision and hearing.

The assessment of visual function included inquiring the respondents about their ability to perceive the presence of a circle on a card and distinguish the break within the circle when illuminated by a flashlight. Four choices could be selected: 1) Can see and distinguish the break in the circle; 2) Can see but can’t distinguish the break in the circle; 3) Can’t see; 4) Blind. Respondents were regarded as having vision impairment if they could not see and distinguish the break in the circle [18].

The interviewers assessed the auditory capacity to ascertain if participants could hear clearly what the interviewers said during the questionnaire survey. Four options are available: 1) Yes, without hearing-aid; 2) Yes, but needs hearing-aid; 3) Partly, despite hearing-aid; 4) No. In accordance with previous research [18], subjects were considered hearing impaired if they could not hear what the interviewers said without a hearing-aid.

According to the results obtained using the aforementioned methodology, the participants were categorized into four distinct groups: 1) no sensory impairment; 2) only visual impairment (VI), without hearing impairment; 3) only hearing impairment (HI), without visual impairment; 4) dual sensory impairment (DSI), with both visual and hearing impairment. Importantly, there was no overlap observed among participants within each respective group [14].

## Assessment of dietary diversity

We employed the dietary diversity score (DDS) to assess the dietary diversity among individuals in the oldest old age group. The frequency of intake for 13 food groups was collected in the CLHLS questionnaire, encompassing fresh fruit, vegetable, meat, fish, eggs, beans, salt-preserved vegetables, sugar, garlic, milk products, nut products, mushroom or algae, and tea. Considering that salt-preserved vegetables and sugar are not recommended according to the dietary guidelines, only the remaining 11 food groups were included in the final calculation of DDS. The survey featured four response options for the frequency of fresh fruit and vegetable consumption: almost every day, often, occasionally, and hardly ever. Respondents were assigned a score of 1 if they answered almost every day or often and 0 if they reported answering occasionally or hardly ever. Frequency of intake of the remaining 9 kinds of food included five options: daily, weekly, monthly, occasionally, and almost never. Respondents were given a score of 1 if they answered daily or weekly and a score of 0 if they answered monthly or occasionally, or almost never. The final DDS of each respondent was obtained by summing all food scores, which ranged from 0 to 11 [30–32]. We divided the degree of dietary diversity (LDD) into two distinct groups: low DDS (< 4) and high DDS (≥ 4) based on the mean of the individuals' scores.

## Assessment of covariates

To control for potential confounding factors, other measures in the CLHLS survey that may have influenced the relationship between diet and sensory issues were included as covariates in the analysis. Sociodemographic variables included age, sex, ethnicity (Han or others), residence (urban, town, or rural), co-residence (living with household members, alone, or in an institution), education (schooling year), economic status (rich, ordinary, or poor). Lifestyle factors included smoking status (current, former, or never), drinking status (current, former, or never), exercise (current, former, or never), sleep quality (good, general, or bad), and sleep duration. Other dietary habits included type of staple food (rice, wheat, or rice and wheat), type of cooking oil (plant oil or animal oil), flavor preference (insipidity and others), intake of vitamins, medicinal plants, and nutrient supplements (yes or no). Physical conditions included self-reported general health status (good, general, or bad) and the number of chronic diseases [33].

## Statistical analysis

The respondents’ characteristics were compared across categories of sensory impairments (visual impairment, hearing impairment, and dual sensory impairment). Continuous variables were presented as mean ± standard deviation (SD), whereas categorical variables were presented as numbers and percentages. To compare the different groups, one-way analysis of variance (ANOVA) followed by a least significant difference (LSD) with post hoc multiple comparisons for continuous variables and the chi-squared test with the Bonferroni correction for categorical variables were used to evaluate differences.

Given potential confounding factors, we constructed a multi-level multiple logistic regression model. Firstly, we conducted a logistic regression for each and all of the 13 food groups in the questionnaire and compared each type of sensory impairment with no sensory impairment. We built five models to evaluate the relationship between sensory impairment and dietary diversity, taking the status of sensory impairment as the dependent variable and DDS with LDD as the independent variable. Analyses were performed as a crude model without any adjustment. Adjusted model 1 was additionally adjusted for sociodemographic characteristics. Adjusted model 2 was additionally adjusted for sociodemographic characteristics and lifestyle factors. Adjusted model 3 was additionally adjusted for sociodemographic information, lifestyle factors, and other dietary habits. Adjusted model 4 was additionally adjusted for sociodemographic information, lifestyle factors, other dietary habits, and physical conditions. The main exposure variable was sensory impairment (categorical variable), with no sensory impairment as the reference category.

Person Mean Imputation in Multiple imputation was adopted to deal with missing data, which was performed on a case-by-case basis, replacing missing values with the mean of available items in the same measurement domain. This method is widely used in questionnaire surveys and is recommended by scholars [34]. All tests were deemed statistically significant at a significance level of *P* < 0.05, using a two-sided approach. All data analyses were conducted using SPSS 25 and GraphPad Prism 9.5.1.

## Results

### Characteristics of samples

Table 1 summarizes the baseline demographic characteristics of the respondents in the 2018 wave of the CLHLS, categorized based on their sensory impairment status. The final study sample had 10,093 participants, with a mean age of 92.29 ± 7.75 years. The individuals who participated in the study and had sensory impairments tended to be older, residing predominantly in rural regions, and had a lower education level than others. The prevalence of visual impairment and dual sensory impairment was higher in females compared to males, whereas no significant gender disparity was observed in hearing impairment. Simultaneously, individuals with sensory impairment consistently exhibited poorer self-reported health status and a higher prevalence of comorbidities. To mitigate the influence of potential confounding factors, the regression model incorporated covariates consisting of variables that exhibited statistically significant differences in baseline data.

**Table 1:** Participants characteristics according to sensory impairment of the oldest old in the Chinese Longitudinal Healthy Longevity Survey (CLHLS), *n* = 10,093

Variables	No sensory impairment	Vision impairment	Hearing impairment	Dual sensory impairment	*P*-value
No. subjects (%)	2771(27.5)	1384(13.7)	2228(22.1)	3710(36.8)	
Age, year	88.02 ± 6.79	90.25 ± 7.19	92.33 ± 7.26	96.21 ± 6.90	** < 0.001**
Sex, n (%)					** < 0.001**
Male	1253(45.2)	531(38.4)	1058(47.5)	1210(32.6)	
Female	1518(54.8)	853(61.6)	1170(52.5)	2500(67.4)	
Residence, n (%)					** < 0.001**
Urban	693(25.0)	295(21.3)	529(23.7)	721(19.4)	
Town	900(32.5)	458(33.1)	755(33.9)	1248(33.6)	
Rural	1178(42.5)	631(45.6)	944(42.4)	1741(46.9)	
Ethnicity, n (%)					0.096
Han	2624(94.7)	1292(93.4)	2123(95.3)	3510(94.6)	
Others	147(5.3)	92(6.6)	105(4.7)	200(5.4)	
Co-residence, n (%)					** < 0.001**
With household members	2072(74.8)	1043(75.4)	1741(78.1)	3030(81.7)	
Alone	587(21.2)	279(20.2)	388(17.4)	508(13.7)	
In an institution	112(4.0)	62(4.5)	99(4.4)	172(4.6)	
Smoking status, n (%)					** < 0.001**
Current	440(15.9)	199(14.4)	383(17.2)	458(12.3)	
Former	383(13.8)	157(11.3)	308(13.8)	340(9.2)	
Never	1948(70.3)	1028(74.3)	1537(69.0)	2912(78.5)	
Drinking status, n (%)					** < 0.001**
Current	311(11.2)	149(10.8)	273(12.3)	381(10.3)	
Former	371(13.4)	176(12.7)	269(12.1)	334(9.0)	
Never	2089(75.4)	1059(76.5)	1686(75.7)	2995(80.7)	
Years of education, n (%)					** < 0.001**
0	1207(43.5)	807(58.3)	1160(52.0)	2603(70.1)	
≥ 1	1564(56.4)	577(41.7)	1068(48.0)	1107(29.9)	
Self-reported health, n (%)					** < 0.001**
Good	1432(51.7)	586(42.3)	1051(47.2)	1267(34.2)	
So-so	1041(37.6)	593(42.8)	908(40.8)	1875(50.5)	
Bad	298(10.8)	205(14.8)	269(12.1)	568(15.3)	
Number of diseases, n (%)					** < 0.001**
0	878(31.6)	433(31.2)	712(31.9)	1407(37.9)	
1	903(32.5)	426(30.7)	639(28.6)	1103(29.7)	
≥ 2	990(35.9)	525(38.1)	877(39.5)	1200(32.4)	

## Associations between sensory impairment and 13 food groups

**Fig. 2:**
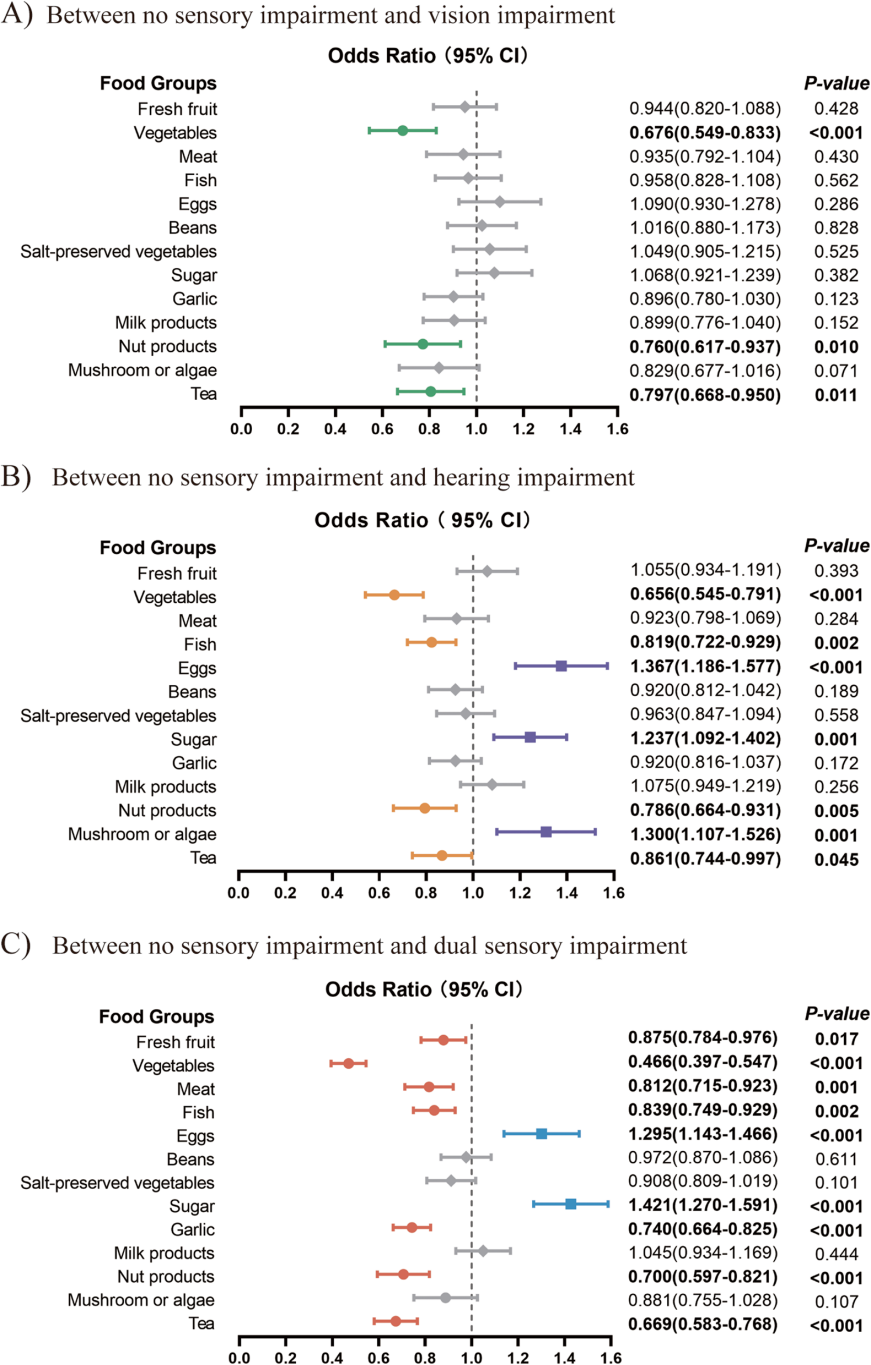
Forest plot of multiple logistic regression of the 13 food groups among each type of sensory impairment*. * abbreviations: CI, confidence interval; Put all 13 groups of food into the multiple logistic regression model simultaneously

The outcomes derived from sequentially inputting the 13 food groups into the model and collectively incorporating all 13 foods are depicted in Fig. 2. The findings from both individual and pooled analyses revealed that the consumption of vegetables (odds ratio [OR] = 0.676, 95% confidence interval [CI], 0.549—0.833), nut products (OR = 0.760, 95%CI, 0.677—0.937), and tea (OR = 0.797, 95%CI, 0.668—0.950) had a protective effect against visual impairment exclusively. Similarly, the regular consumption of vegetables (OR = 0.656, 95%CI, 0.545—0.791), nut products (OR = 0.786, 95%CI, 0.664—0.931), and tea (OR = 0.861, 95%CI, 0.744—0.997) exhibited a protective effect against hearing impairment. Conversely, the consumption of eggs (OR = 1.367, 95%CI, 1.186—1.577), sugar (OR = 1.237, 95%CI, 1.092—1.402), and mushroom or algae (OR = 1.300, 95%CI, 1.107—1.526) were identified as risk factors. Concerning dual sensory impairment, the consumption of fresh fruit (OR = 0.875, 95%CI, 0.784—0.976), vegetables (OR = 0.466, 95%CI, 0.397—0.547), meat (OR = 0.812, 95%CI, 0.715—0.923), fish (OR = 0.839, 95%CI, 0.749—0.929), garlic (OR = 0.740, 95%CI, 0.664—0.825), nut products (OR = 0.700, 95%CI, 0.597—0.821), and tea (OR = 0.669, 95%CI, 0.583—0.768) have been observed to be associated with reduced risk. Conversely, sugar consumption (OR = 1.421, 95%CI, 1.270—1.591) appears to have an adverse effect.

## Associations between sensory impairment and dietary diversity

The dietary diversity indicators encompass two distinct measures: the DDS and the dietary diversity categorization. The findings from the inter-group comparison revealed a statistically significant distinction between the two indicators in the groups without sensory impairment compared to those with vision impairment only (Bonferroni-corrected *P* < 0.001) and without sensory impairment compared to those with dual sensory impairment (Bonferroni-corrected *P* < 0.001). However, no significant difference was observed between the group without sensory impairment and the group with hearing impairment only (Bonferroni-corrected *P* = 0.138).

To further enhance the understanding of the relationship between sensory impairment and dietary diversity, we conducted a reanalysis that considered covariates using the multiple logistic regression model (Table 2). The initial crude model revealed a significant inverse association between DDS and visual impairment (odds ratio [OR] = 0.915, 95% confidence interval [CI], 0.890—0.941), as well as dual sensory impairment (OR = 0.872, 95%CI, 0.853—0.891). In other words, the higher the DDS score, the lower the likelihood of experiencing visual impairment. After controlling for sociodemographic characteristics, lifestyle factors, other dietary habits, and physical conditions, we observed a persistent negative association between DDS and the occurrence of vision impairment in Adjusted model 4: odds ratio [OR] = 0.944 (95% confidence interval [CI], 0.915—0.974), as well as dual sensory impairment in Adjusted model 4: OR = 0.930 (95% CI, 0.905—0.955). Nevertheless, our results indicated that there is no significant correlation between dietary diversification and the prevalence of hearing impairment.

**Table 2:** Logistic regression analysis between dietary diversity and sensory impairment^a^

	**Sensory impairment**	**Dietary diversity score (DDS)**		**Level of dietary diversity** **(DDS ≥ 4 vs. DDS < 4)**	
		**OR (95% CI)**	***P*-value**	**OR (95% CI)**	***P*-value**
	No sensory impairment	1(Reference)		1(Reference)	
	Vision impairment				
**Crude model**		**0.915(0.890–0.941)**	** < 0.001**	**0.727(0.638–0.829)**	** < 0.001**
**Adjusted model 1**		**0.935(0.907–0.9764)**	** < 0.001**	**0.787(0.686–0.903)**	** < 0.001**
**Adjusted model 2**		**0.951(0.922–0.981)**	**0.002**	**0.841(0.731–0.967)**	**0.015**
**Adjusted model 3**		**0.948(0.918–0.978)**	** < 0.001**	**0.828(0.720–0.953)**	**0.008**
**Adjusted model 4**		**0.944(0.915–0.974)**	** < 0.001**	**0.820(0.713–0.944)**	**0.002**
	No sensory impairment	1(Reference)		1(Reference)	
	Hearing impairment				
**Crude model**		0.985(0.962–1.009)	0.213	0.895(0.798–1.004)	0.059
**Adjusted model 1**		0.999(0.972–1.026)	0.919	0.921(0.814–1.041)	0.188
**Adjusted model 2**		1.005(0.978–1.032)	0.737	0.937(0.828–1.062)	0.309
**Adjusted model 3**		1.004(0.977–1.032)	0.776	0.937(0.827–1.062)	0.309
**Adjusted model 4**		0.998(0.971–1.026)	0.909	0.924(0.815–1.047)	0.214
	No sensory impairment	1(Reference)		1(Reference)	
	Dual sensory impairment				
**Crude model**		**0.872(0.853–0.891)**	** < 0.001**	**0.598(0.541–0.661)**	** < 0.001**
**Adjusted model 1**		**0.913(0.890–0.937)**	** < 0.001**	**0.698(0.622–0.783)**	** < 0.001**
**Adjusted model 2**		**0.938(0.913–0.962)**	** < 0.001**	**0.771(0.685–0.867)**	** < 0.001**
**Adjusted model 3**		**0.935(0.910–0.960)**	** < 0.001**	**0.762(0.677–0.858)**	** < 0.001**
**Adjusted model 4**		**0.930(0.905–0.955)**	** < 0.001**	**0.751(0.667–0.846)**	** < 0.001**

^a^Adjusted model 1 adjusted for age, sex, ethnicity, residence, co-residence, education and economic status. Adjusted model 2 further adjusted for smoking status, drinking status, exercise, sleep quality and sleep duration. Adjusted model 3 further adjusted for type of staple food, type of cooking oil, flavor preference, intake of vitamins, medicinal plants and nutrient supplements. Adjusted model 4 further adjusted for self-reported health status and the number of co-chronic diseases

Moreover, when examining the crude model, it was observed that individuals with high dietary diversity levels had lower OR for vision impairment (OR = 0.727, 95% CI: 0.683—0.829) and dual sensory impairment (OR = 0.598, 95% CI: 0.541—0.661) compared to those with low dietary diversity. These findings suggest that older adults with low dietary diversity are at a higher risk of experiencing vision and dual sensory impairment than those with high dietary diversity. After adjusting for sociodemographic characteristics, lifestyle factors, other dietary habits, and physical conditions, the odds ratio (OR) of individuals with high dietary diversity for experiencing vision impairment in Adjusted model 4 was found to be 0.820 (95% CI, 0.713—0.944). Similarly, the odds ratio for dual sensory impairment was 0.751 (95% CI, 0.667—0.846). Furthermore, when comparing individuals with low dietary diversity to those with high dietary diversity, it was found that older adults with high dietary diversity did not exhibit a reduced incidence of hearing impairment in all models. The OR for this association was calculated as 0.895 (95% CI, 0.798—1.004), 0.921 (95% CI, 0.814—1.041), 0.937 (95% CI, 0.828—1.062), 0.937 (95% CI, 0.827—1.062), and 0.924 (95% CI, 0.851—1.047), respectively.

## Discussion

The prevalence of age-related visual and auditory impairment is experiencing a sharp rise due to the growing trend of population aging, specifically among the oldest old. However, this particular group has yet to garner significant attention in previous research. The WHO is committed to the advancement of healthy aging between the years 2015 and 2030, defining it as the sustenance of functional capacity, which facilitates overall well-being during older adulthood, including vision and auditory health [35]. The study revealed that diversified, well-balanced, and nutritionally-rich dietary patterns possess the potential to retard aging progression and facilitate healthy aging through various underlying mechanisms [28]. Sensory impairments constitute an inherent and significant component of the aging process. Hence, it is plausible to posit that dietary adjustment can potentially decrease both the occurrence and severity of these illnesses [26, 36, 37].

In this current investigation, we utilized a substantial dataset comprising one of the most extensive collections of the oldest old populations in China. It was shown that vision, hearing, and dual sensory impairment were prevalent problems among the oldest old population in China, typically among those who were older, had lower levels of education, and resided in rural regions. Interestingly, our findings suggest a higher prevalence of visual impairment and dual sensory impairment in females compared to males, while no significant gender disparity was observed regarding hearing impairment. The observed differences can be ascribed to biological reasons, including the intrinsic disparity in lifespan between women and men, as well as the heightened vulnerability of women to specific visual conditions such as cataracts [8, 38]. Regarding the association between gender and hearing impairment prevalence rates, previous research has indicated a higher prevalence rate among males compared to females [39]. A Chinese population-based survey revealed that the incidence of hearing disabilities, such as otitis media, and drug-induced toxicity, is higher in males compared to females. Conversely, the prevalence of systemic diseases leading to hearing impairment is greater among females than males. Furthermore, no significant gender disparity was observed in cases of age-related deafness [40]. In addition to the physiological changes that occur naturally with aging, sensory functions are intricately linked to other pathological diseases that are associated with advancing age [41–43]. Moreover, socioeconomic factors, such as limited educational attainment, inadequate disease awareness and knowledge, and insufficient healthcare accessibility in rural areas, are also likely contributing factors [44]. Therefore, it is imperative to prioritize the vision and hearing impairments of elderly individuals. By focusing on a modifiable lifestyle risk factor, namely a healthy and varied diet, it is possible to reduce the likelihood of vision and hearing impairments, subsequently maintaining optimal organ and sensory function throughout the aging process [36].

The present study underscored the importance of a diverse and well-rounded diet in effectively mitigating the risk of developing visual impairment and dual sensory impairment in the oldest old. On the one hand, substantial evidence appeals to a diet rich in vegetables, nuts, fruits containing high levels of vitamins C and E, as well as carotenoids lutein and zeaxanthin. These interventions can reduce the risk of age-related macular degeneration (AMD), specific subtypes of cataracts, glaucoma, and other age-related visual diseases [45–52]. On the other hand, many studies have revealed that increased consumption of fish, long-chain polyunsaturated fatty acids (PUFAs), folate, beta-carotene, and vitamins A, E, and C is associated with reduced risk ratios for hearing loss [53, 54]. Collectively, it can be posited that the implementation of a nutritionally balanced and varied diet, holds the potential to reduce the risk of developing visual and auditory deficits in older adults.

Regarding dietary determinants, our findings revealed a significant association between regular intake of vegetables, nut products, and tea with a reduced likelihood of sensory disorders. According to the National Health and Nutrition Examination Study (NHANES), adopting a high-quality diet like the Mediterranean diet, which consists of nutrient-dense vegetables, fruits, fish, olive oil, and moderate wine consumption, can provide significant amounts of antioxidants and micro-nutrients [55–57]. These components have been linked to regulating metabolic conditions associated with inflammation and oxidative stress [23, 58]. Though there appear to be etiological distinctions among visual and auditory disorders, it is essential to note the parallels in their pathophysiology. The occurrence of these illnesses was partly initialized by the presence of oxidative stress and chronic inflammation, which were identified as key variables [45, 59–61]. Therefore, a diversified diet rich in vegetables, fruits, and high-quality proteins may play a protective role in reducing sensory deficits.

### Limitations

It is important to acknowledge several limitations that exist within our study. Initially, it should be noted that several information was based on self-report rather than quantitative measurements. Therefore, the measurements may be affected by several factors, such as cognitive abilities, educational background, memory bias, and personal characteristics. Furthermore, although our study based on a cross-sectional setting, it provides a reliable representation of the daily dietary habits among the older age group. Hence, the findings of this study demonstrated a noteworthy association, underscoring the prevention potential. Future investigations will incorporate longitudinal cohorts to further elucidate the underlying causal relationship between the dietary factors and sensory dysfunction. The omission of the supplementary nutritional products from the study was attributed to a substantial quantity of incomplete data. The correlation between distinct nutrients and sensory impairment has yet to be fully understood.

## Conclusion and implications

In conclusion, the present study presents findings that support the notion that increased dietary diversity is associated with a decreased likelihood of experiencing vision and dual sensory impairment, as indicated by a nationally representative sample. Furthermore, the regular consumption of vegetables, nut products, and tea exhibited an inverse correlation with the incidence of sensory impairments in the oldest old Chinese population. The synthesis of research findings suggests that following diverse dietary patterns and healthy nutritional practices may be an effective and affordable way to prevent age-related decline in audio-visual capabilities.

### Role of the funder

The funders had no role in the design and conduct of the study; collection, management, analysis, and interpretation of the data; preparation, review, or approval of the manuscript; and decision to submit the manuscript for publication.

## Data Availability

Data described in the manuscript, code book, and analytic code will be made publicly and freely available without restriction at http://opendata.pku.edu.cn/.
